# Road safety assessment and risks prioritization using an integrated SWARA and MARCOS approach under spherical fuzzy environment

**DOI:** 10.1007/s00521-022-07929-4

**Published:** 2022-10-25

**Authors:** Saeid Jafarzadeh Ghoushchi, Sina Shaffiee Haghshenas, Ali Memarpour Ghiaci, Giuseppe Guido, Alessandro Vitale

**Affiliations:** 1 Faculty of Industrial Engineering, Urmia University of Technology, Urmia, Iran; 2 Department of Civil Engineering, University of Calabria, Via Bucci, 87036 Rende, Italy

**Keywords:** FMEA, Spherical fuzzy sets, SWARA, MARCOS, Road safety, Sustainable development, Sustainable mobility

## Abstract

There are a lot of elements that make road safety assessment situations unpredictable and hard to understand. This could put people's lives in danger, hurt the mental health of a society, and cause permanent financial and human losses. Due to the ambiguity and uncertainty of the risk assessment process, a multi-criteria decision-making technique for dealing with complex systems that involves choosing one of many options is an important strategy of assessing road safety. In this study, an integrated stepwise weight assessment ratio analysis (SWARA) with measurement of alternatives and ranking according to compromise solution (MARCOS) approach under a spherical fuzzy (SF) set was considered. Then, the proposed methodology was applied to develop the approach of failure mode and effect analysis (FMEA) for rural roads in Cosenza, southern Italy. Also, the results of modified FMEA by SF-SWARA-MARCOS were compared with the results of conventional FMEA. The risk score results demonstrated that the source of risk (human) plays a significant role in crashes compared to other sources of risk. The two risks, including landslides and floods, had the lowest values among the factors affecting rural road safety in Calabria, respectively. The correlation between scenario outcomes and main ranking orders in weight values was also investigated. This study was done in line with the goals of sustainable development and the goal of sustainable mobility, which was to find risks and lower the number of accidents on the road. As a result, it is thus essential to reconsider laws and measures necessary to reduce human risks on the regional road network of Calabria to improve road safety.

## Introduction

Failure mode and effect analysis (FMEA) is a common risk analysis method used to find and fix possible failures, problems, and mistakes in a process, system, design, or service in order to make it safer and more reliable [1]. System safety is enhanced when FMEA aims to recognize all potential risk factors in a system, prioritize the potential risk factors, and take corrective actions to eliminate or decrease the high-risk ones. FMEA unlike other critical analysis techniques is a preventive tool that discover a solution before failure occurs [2]. Therefore, it can prepare valuable information for risk managers, guide them to modify existing plans, take estimates to decrease the possibility of failure, and avoid dangerous accidents. A cross-functional team should be formed first before analyzing a particular system or product. This will allow an FMEA to be carried out. Then, FMEA team members should determine all potential product or system failure modes [3].

The risk factors prioritization is evaluated with Risk Priority Number (RPN) by employing Severity (S), Occurrence (O), and Detection (D) [4], which is defined as Eq. (1). Each parameter takes values as 1 lowest and 10 highest.

1 $$\mathrm{RPN}=\mathrm{S}\times \mathrm{O}\times \mathrm{D}$$

Risk factors with higher RPN are more significant and will require incite activities to avoid or relieve probable risks [5]. Though a systematic way to rank risk assessments, this conventional FMEA logic has some disadvantages. The following shortcomings can be attributed to conventional FMEA:

(A) Apart from triple risk factors (S, O, and D), additional parameters like Cost (C) that have an impact on risk assessment have not been considered [6, 7].
(B) Weights of S, O, and D are not considered when computing RPN in conventional FMEA [8, 9].
(C) It is challenging to examine precisely the S, O, and D parameters due to their subjective evaluation on a 1–10 scale. Using linguistic terms in fuzzy numbers, the FMEA can be better driven [6, 9, 10].
(D) The RPN elements have duplicate numbers. Sometimes two or more risk factors may have same RPN scores. Conventional FMEA does not thoroughly prioritize risk assessments and confuses risk management decision-makers [11].

In this study, an adjusted FMEA model based on the spherical fuzzy extension of Measurement of Alternatives and Ranking according to COmpromise Solution (SF-MARCOS) is proposed to overcome the conventional RPN assessment limitations style.

The fuzzy set theory was introduced by Lotfi [12] to overcome ambiguity and uncertainty in difficult situations to determine whether the crisp state is true or false, which is not a clear frontier of information. Since then, fuzzy sets have been used in many research fields [13, 14], and the theory has been extended to have more reliable results. SFS has recently been utilized in several applications, such as landfill site selection for medical waste [15], waste disposal location selection [16], and design evaluation and technology of a linear delta robot [17]. Ashraf et al. [18] developed and applied spherical fuzzy operators to Dombi aggregation to solve group decision-making problems and used spherical fuzzy to detect the cov-19. Also, spherical fuzzy sets are applied in multi-criteria issues. Kutlu Gündoğdu and Kahraman [16] presented the spherical fuzzy VIKOR (SF-VIKOR) method to show spherical fuzzy applicability. They also extended the conventional WASPAS method to the spherical fuzzy WASPAS (SF-WASPAS) method [19]. Jafarzadeh Ghoushchi et al. [11] applied spherical fuzzy MOORA to prioritize circular economy implementation barriers in the designing of sustainable medical waste management systems. Boltürk [20] applied spherical fuzzy TOPSIS and compared the results with the neutrosophic TOPSIS method. Gündoğdu and Kahraman [17] proposed a spherical fuzzy QFD (SF-QFD) method that includes linguistic assessment under certainty and uncertainty.

Nevertheless, the MARCOS technique has not been merged with the SWARA method based on the FMEA technique within the context of SFSs, though SFSs have been proven as one of the valuable tools to handle the uncertainty and vagueness that occur in real-life concerns. Consequently, the present study focuses on SFSs. This type of fuzzy set eliminates some aspects of neutrosophic sets and Pythagorean fuzzy sets by requiring the sum of membership, non-membership, and hesitancy degrees not to exceed one. It does not disregard independent hesitancy, in contrast to Pythagorean fuzzy sets. Even though many researchers have focused on the SFS context, no one has looked at the serious risks that could be present in rural road prioritization problems in a spherical fuzzy environment. Existing literature shows that there is a need to evaluate the critical potential risk factors in rural roads because the evaluation of critical potential risk is not only a vital issue but also an uncertain subject [21–24]. The rural roads’ critical potential risk factors selection process includes many objective and subjective attributes with conflicting goals. Thus, the above problem requires a systematic and suitable approach to evaluate rural roads' critical potential risk factors. To address this concern, an integrated SF–SWARA-MARCOS method is developed that allows DMs to specify a membership function on a spherical area to generalize other fuzzy set components and assign the membership performance parameters independently of the larger domain. Hence, the developed approach can provide more relevant and accurate results by utilizing the advantage of the SFS set, which reflects uncertainty more appropriately and is equivalent to judgments made by decision-makers in assessing potential risks on rural roads. The main contributions of this study are as follows:

(A) Provide an FMEA based SF-SWARA-MARCOS integration framework
(B) Consider Cost (C) as an essential management indicator with SOD factors
(C) Using the SFS-based SWARA to evaluate criteria weights
(D) Presenting and evaluating a real problem in prioritizing the existing potential severe risk factors in rural roads of the Calabria region (Italy) using the proposed method SF-SWARA-MARCOS and showing the application of the introduced method
(E) Sensitivity and comparison analysis to validate and reveal the usefulness of the suggested method

The rest of this research is structured as follows: In Sect. 2, several researches are investigated in three sub-sections as FMEA model and methods based on it, applications of SWARA and MARCOS methods separately. In Sect. 3, additional descriptions of Spherical fuzzy sets and SF-SWARA are provided. In Sect. 4, extended Spherical fuzzy MARCOS (SF-MARCOS) is introduced. Then, the suggested approach to this study is presented. Finally, a case study is introduced, and the results from implementing the suggested approach are analyzed in Sect. 4. In the end, the conclusions and evolution suggestions of this research are presented in Sect. 5.

## Literature review

### Hybrid FMEA approach

Failure mode and effect analysis (FMEA) is a method widely utilized in different industries to detect potential failures. Reliability analysis is increasing in today's complex systems to find and prevent probable failures to improve the reliability of products, processes, safety, and systems. Apart from different reliability management tools, the main goal of the failure mode and effect analysis (FMEA) is to identify and prioritize the potential failure modes before a failure arises [25]. Failure modes with a higher RPN are more important and need to be fixed quickly to avoid or reduce possible risks [5]. FMEA has been used in various fields due to its application. The tunnel construction process is a very costly and risky project built in uncertain conditions, and there may be risks in the tunnel construction process. Therefore, Amini and Mojtaba [26] have used the FMEA technique to identify, classify, and analyze potential hazards in the tunnel construction process. The vehicle, the environment, and the car's driver are the three elements associated with driving, and the failure of each leads to an accident. By examining potential errors, effects, and causes, solutions can be offered to prevent them. For this reason, the FMAE technique has been used to analyze driving errors [27]. The traditional RPN index used in this method suffers from significant drawbacks, such as allocating the same weight to different risk factors and an absence of complete ranking in the risk evaluation procedure [9]. Several multiple criteria decision-making (MCDM)-based FMEA have been proposed [28]. Moreover, the literature has made many efforts to improve FMEA using fuzzy inference. FMEA has been used to identify significant human errors, causes, and effects during Computer numerical control (CNC) machining operations. The Analytic Hierarchy Process (AHP) method is used to calculate the weight of risk factors, and the Measurement of Alternatives and Ranking according to the compromise Solution (MARCOS) method is used to rank the risks. In addition, a Fuzzy environment has been used to reduce the uncertainty in the data [29]. Also, due to the shortcomings of the FMEA method, Alvand, Mirhosseini et al. [30] have used the integrated Stepwise Weight Assessment Ratio Analysis (SWARA) and Weighted Aggregated Sum Product Assessment (WASPAS) approaches in a fuzzy environment in order to identify and rank the risks in construction projects. Finally, the results show that the combination of MCDM and FMEA methods is more capable and accurate than conventional FMEA. Also, a combination of MCDM and FMEA methods in a fuzzy environment has been used to identify risks in the automotive spare parts industry. In this study, the Best–Worst Method (BWM) method is used to weight the criteria, and the Multi-Objective Optimization by Ratio Analysis (MOORA) method is used to prioritize the risks [31].

### SWARA applications

The stepwise weight assessment ratio analysis (SWARA) approach was introduced by Keršuliene et al. [32], to appointing suitable weights to the criteria. In this method, decision-makers (DMs) have an important role in assessing the criteria weights. At first, each DM prioritizes the criteria according to their significance [33]. The major attribute of the SWARA method is that DMs’ views about the importance ratio of the criteria are approximated through the weights find out process. The capability to evaluate experts’ opinion about significance ratio of the criteria in the process of their weights calculation is the main element of the SWARA method. Furthermore, SWARA is helpful for gathering data from experts. Moreover, SWARA method is uncomplicated and the experts can comfortably work together [34]. SWARA can be applied in many complicated MCDM problems, such as selection of supplier [35], packaging design [36], selection of machine tool [37], logistic problems [38], and selection of personnel [39, 40]. Moreover, SWARA is a beneficial method where issues ranks are already clarified and criteria assessment is not needed anymore, while other criteria weighting methods, like the Analytic Hierarchy Process (AHP), are highly related to the criteria assessments. A SWARA-COPRAS method was recommended by Hashemkhani Zolfani and Bahrami [41] for prioritizing high-tech industries investments. Vojinović et al. [42] applied a novel approach include IMF SWRA method to assess healthcare system. A SWARA-WASPAS approach has been proposed to solve a problem of location selection in turkey [43].

Most of the time, DMs’ judgments are not express because of hesitancy and vagueness of their views or lack of information; thus, to manage this issue, researchers have extended MCDM methods in uncertain environments [44, 45]. A hybrid fuzzy SWARA-COPRAS approach was proposed by Zarbakhshnia et al. [46], to assess and select the sustainable provider. Ghoushchi et al. [47] applied developed SWARA and CoCoSo methods to evaluate wind turbine failure modes. A Pythagorean fuzzy SWARA and VIKOR framework was recommended in another study to choose the solar panel performance assessment [48]. Ghoushchi et al. [49] proposed a hybrid Z-SWARA and Z-MOORA approach with a developed FMEA method to cope some shortcomings of traditional FMEA. An extended interval-valued PF- SWARA-MULTIMOORA was presented by He et al. [50] to examine the status of tourism community in India. Ghoushchi et al. [15] proposed an integrated SF-SWARA-WASPAS approach to select the suitable location for medical waste landfill site. Rahmati et al. [51] discussed a new methodology combining Z-SWARA and Z-WASPAS techniques with the FMEA method to evaluate the priority of risk factors of financial measurement for production companies' management control systems.

### MARCOS applications

MARCOS is a new MCDM method introduced by Stević et al. [52]. MARCOS accelerates decision-making in the literature by solving a wide range of different problems. Defining the relationship between the options and the ideal and counter-ideal degrees as reference points determines the utility functions of the options and obtains an adaptive ranking of the options. Higher efficiency, ease in structuring and optimizing the decision process, more accurate determination of the degree of desirability concerning the reference point, more excellent stability and robustness of the results in terms of changing measurement scales, and no problem of ranking inversion [52] are some of the advantages of MARCOS method compared to other MCDM methods such as WASPAS, SAW, and TOPSIS. The MARCOS method has been used in various fields. In this regard, Stević and Brković [53] used the MARCOS method to evaluate the transportation system of an international transportation company. They used this method to rank 25 drivers based on five criteria. Finally, a sensitivity analysis was performed between MARCOS methods and other methods, which shows the results of the superiority of MARCOS method and the reliability of the ranking. In addition, the MARCOS method has also been used to evaluate battery-powered electric vehicles. In this work, 10 cars have been selected as alternatives and have been ranked based on technical specifications such as price, battery, energy, and allowable load. Sensitivity analysis has also been performed to show the validity and robustness of the results [54]. Chakraborty et al. [55] applied a modified MARCOS technique with the application of D numbers to select suitable supplier. Also, an integrated F-PIPRECIA and F-MARCOS have been used in regional aircraft selection problem [56].

MCDM methods allow experts to select the best option based on multiple criteria, the relative importance of the criteria, qualitative and quantitative information, and the preferences of DMs [57]. On the other hand, the opinions and preferences of experts are usually vague and inaccurate when evaluating and selecting options based on various criteria, so the fuzzy concept is combined with MCDM methods to consider the uncertainty. For these reasons, Stanković et al. [58] have developed the MARCOS method in a fuzzy environment. MARCOS has been used to get rid of ambiguity and uncertainty in a number of different subjects. For example, to evaluate the quality of electronic services in the aviation industry from the perspective of consumers, the AHP and MARCOS methods have been used. Due to the ambiguous nature of e-services evaluation, the fuzzy concept has been used. First, the three criteria of reliability, security, and comprehensibility were weighed by the Fuzzy AHP method, and then, the ranking was done by the Fuzzy MARCOS method [59]. Ecer and Pamucar [60] have used the MARCOS method in an intuitionistic fuzzy environment to evaluate and rank insurance companies in healthcare services.

To date, there are few studies that have investigated the applications and capabilities of MARCOS method in uncertain environments. As a result, based on the literature, there is no study reported to evaluate rural roads' critical potential risk factors by applying FMEA-based methodology in the context of spherical fuzzy. Hence, it motivated us to focus on this topic and propose a modified SF-SWARA-MARCOS approach base on the FMEA technique as a contribution of this study.

### Prelimination of spherical fuzzy sets

The concept of spherical fuzzy set (SFS) is one of the latest fuzzy sets proposed by Kutlu Gündoğdu and Kahraman [61]. Zadeh [62] introduced Type-2 fuzzy sets; compared to the membership function of type-1 fuzzy sets, which are two-dimensional and fixed, type-2 fuzzy sets have a fuzzy three-dimensional membership function that provides more freedom to the decision-making process [63]. Intuitionistic fuzzy set (IFS) is a two-dimensional that defines both membership and non-membership degrees. Atanassov [64] evolved intuitionistic fuzzy sets (IFSs) with the aid of introducing indices, membership degree (*μ*(*x*)), and non-membership degree ($\upsilon$(*x*)) such that 0 ≤ *µ*(*x*) + $\upsilon$(*x*) ≤ 1. Yager [65] developed the Pythagorean fuzzy set (PFS) such that 0 ≤ ${\mu }^{2}$(*x*) + $\upsilon^{2}$(*x*) ≤ 1. It is a generalization of IFSs. Finally, the Spherical fuzzy set (SFS) was proposed by Kutlu Gündoğdu and Kahraman [61], which is the generalized form of Neutrosophic sets (NSs), PFSs, and picture fuzzy sets (PiFSs). Spherical sets offer an innovative approach to a selection of problems that have historically proven very difficult to resolve through existing other extensions of fuzzy set theory, such as human suppositions, including answers such as yes, no, abstain, and refusal. According to Ashraf et al. [66], the main difference between PFS and SFS is that in SFS, we study a neutral degree, while in PFS, it does not [67, 68]. This section presents some of the properties, arithmetic operations, and principles of SFSs.

#### Definition 1

Spherical Fuzzy Sets (SFS) *S *of the universe of discourse *X* is given by:

2 $$ S = \left[ {\left( {x.\left( {\mu_{S} \left( x \right).v_{S} \left( x \right).\pi_{S} \left( x \right)} \right)} \right)|x\, \in \,X} \right] $$

where $${\mu }_{S}:X\to \left[0.1\right].{v}_{S}:X\to \left[0.1\right].{\pi }_{S}:X\to \left[0.1\right]$$

For each *x*, the numbers ${\mu }_{S}$, ${v}_{S}$, and ${\pi }_{S}$ represent the degrees of membership, non-membership, and hesitance for every $x\, \in \,X$ in the SFS $S$, respectively. Also,

3 $$0\le {\left({\mu }_{{s}}\left(x\right)\right)}^{2}+{\left({v}_{{s}}\left(x\right)\right)}^{2}+{\left({\pi }_{{s}}\left(x\right)\right)}^{2}\le 1$$

#### Definition 2

Let ${{A}}_{S}=\left[{\mu }_{{{A}}_{S}}.{v}_{{{A}}_{S}}.{\pi }_{{{A}}_{S}}\right]$ and ${{B}}_{S}=\left[{\mu }_{{{B}}_{S}}.{v}_{{{B}}_{S}}.{\pi }_{{{B}}_{S}}\right]$ be two SFSs and ${k}$ is greater than 0 as a constant number. Basic mathematical operations are defined as given as follows:

4 $${{A}}_{S}\oplus {{B}}_{S}=\left[\sqrt{{\mu }_{{{A}}_{S}}^{2}+{\mu }_{{{B}}_{S}}^{2}-{\mu }_{{{A}}_{S}}^{2}{\mu }_{{{B}}_{S}}^{2}} .{v}_{{{A}}_{S}}{v}_{{{B}}_{S} }.\sqrt{\left(1-{\mu }_{{{B}}_{S}}^{2}\right){\pi }_{{{A}}_{S}}+\left(1-{\mu }_{{{A}}_{S}}^{2}\right){\pi }_{{{B}}_{S}}-{\pi }_{{{A}}_{S}}}{\pi }_{{{B}}_{S}}\right]$$

5 $${{A}}_{S}\otimes {{B}}_{S}=\left[{\mu }_{{{A}}_{S}}{\mu }_{{{B}}_{S}}.\sqrt{{v}_{{{A}}_{S}}^{2}+{v}_{{{B}}_{S}}^{2}-{v}_{{{A}}_{S}}^{2}{v}_{{{B}}_{S}}^{2}} .\sqrt{\left(1-{v}_{{{B}}_{S}}^{2}\right){\pi }_{{{A}}_{S}}^{2}+\left(1-{v}_{{{A}}_{S}}^{2}\right){\pi }_{{{B}}_{S}}^{2}-{\pi }_{{{A}}_{S}}^{2}{\pi }_{{{B}}_{S}}^{2}}\right]$$

6 $${kS}=\left[\sqrt{1-{\left(1-{\mu }_{{s}}^{2}\right)}^{{k}} }.{v}_{{s}}^{{k}}.\sqrt{{\left(1-{\mu }_{{s}}^{2}\right)}^{{k}}-{\left(1-{\mu }_{{s}}^{2}-{\pi }_{{s}}^{2}\right)}^{{k}}}\right]$$

7 $${S}^{{k}}={\mu }_{{s}}^{{k}}.\sqrt{1-{\left(1-{v}_{{s}}^{2}\right)}^{{k}}} .\sqrt{{\left(1-{v}_{{s}}^{2}\right)}^{{k}}-{\left(1-{v}_{{s}}^{2}-{\pi }_{{s}}^{2}\right)}^{{k}}}$$

#### Definition 3

For these SFS ${{A}}_{S}=\left[{\mu }_{{{A}}_{S}}.{v}_{{{A}}_{S}}.{\pi }_{{{A}}_{S}}\right]$ and ${{B}}_{S}=\left[{\mu }_{{{B}}_{S}}.{v}_{{{B}}_{S}}.{\pi }_{{{B}}_{S}}\right],$ These rules under the condition ${{k},{ k}}_{1}, {{k}}_{2}>0 ,$ are valid:

8 $${{A}}_{S}\oplus {{B}}_{S}={{B}}_{S}\oplus {{A}}_{S}$$

9 $${{A}}_{S}\otimes {{B}}_{S}= {{B}}_{S}\otimes {{A}}_{S}$$

10 $${k}\left({{A}}_{S}\oplus {{B}}_{S}\right)={{kA}}_{S}\oplus {{kB}}_{S}$$

11 $${{k}}_{1}{{A}}_{S}+{{k}}_{2}{{A}}_{S}=\left({{k}}_{1}+{{k}}_{2}\right){{A}}_{S}$$

12 $${({{A}}_{S}\otimes {{B}}_{S})}^{{k}}={{{A}}_{S}}^{{k}}\otimes {{{B}}_{S}}^{{k}}$$

13 $${{{A}}_{S}}^{{k}1}\otimes {{{A}}_{S}}^{{k}2}={{{A}}_{S}}^{{k}1+{k}2} $$

#### Definition 4

The score value (SV) and accuracy function (AF) of the number $S=${${\mu }_{{s}}.{\upnu }_{{s}}.{\pi }_{{s}}$} are calculated as follows:

14 $${Score }\left({S}\right)={\left({\mu }_{{s}}-{\pi }_{{s}}\right)}^{2}-{\left({v}_{{s}}-{\pi }_{{s}}\right)}^{2} $$

15 $$\mathrm{Accuracy}\left({S}\right)={\mu }_{{s}}^{2}+{v}_{{s}}^{2}+{\pi }_{{s}}^{2} $$

Note that: ${{A}}_{S}<{{B}}_{S}$ if and only if

16 $$ \begin{array}{*{20}l} {{\text{i}}{.}} \hfill & {\quad {\text{score }}\left( {A_{S} } \right) < {\text{score}} \left( {B_{S} } \right) \,{\text{or}}} \hfill \\ {{\text{ii}}{.}} \hfill & {\quad {\text{score }}\left( {A_{S} } \right) = {\text{score}} \left( {B_{S} } \right) \,{\text{and}} \,{\text{Accuracy}} \left( {A_{S} } \right) < {\text{Accuracy}} \left( {B_{S} } \right)} \hfill \\ \end{array} $$

#### Definition 5

Spherical Weighted Arithmetic Mean (SWAM) with respect to, $w = (w_{1} .w_{2}  \ldots w_{n} ).w_{i} \, \in \,[0,1];\sum\nolimits_{{(i = 1)}}^{n} {w_{i}  = 1} ,$ is computed as follows:

17 $$ \begin{aligned} & {\text{SWAM}}_{w} \left( {S_{1} \ldots .S_{n} } \right) = w_{1} S_{1} + w_{2} S_{2} + \cdots + w_{n} S_{n} \\ & \quad = \left\{ {\left[ {1 - \mathop \prod \limits_{i = 1}^{n} \left( {1 - \mu_{s}^{2} } \right)^{wi} } \right]^{\frac{1}{2}} . \mathop \prod \limits_{i = 1}^{n} v_{s}^{wi} .\left[ {\mathop \prod \limits_{i = 1}^{n} \left( {1 - \mu_{s}^{2} } \right)^{wi} - \mathop \prod \limits_{i = 1}^{n} \left( {1 - \mu_{s}^{2} - \pi_{s}^{2} } \right)^{wi} } \right]^{\frac{1}{2}} } \right\} \\ \end{aligned} $$

#### Definition 6

Spherical Weighted Geometric Mean (SWGM) with respect to, $w = (w_{1} .w_{2} \ldots w_{n} ).w_{i} \, \in \,[0.1];\sum\nolimits_{(i = 1)}^{n} {w_{i} = 1} ,$ is computed as follows:

18 $$ \begin{aligned} & {\text{SWGM}}_{w} \left( {S_{1} \ldots .S_{n} } \right) = S_{1}^{w1} + S_{2}^{w2} + \cdots + S_{n}^{wn} \\ & \quad = \left\{ {\mathop \prod \limits_{i = 1}^{n} \mu_{s}^{wi} . \left[ {1 - \mathop \prod \limits_{i = 1}^{n} \left( {1 - v_{s}^{2} } \right)^{wi} } \right]^{\frac{1}{2}} .\left[ {\mathop \prod \limits_{i = 1}^{n} \left( {1 - v_{s}^{2} } \right)^{wi} - \mathop \prod \limits_{i = 1}^{n} \left( {1 - v_{s}^{2} - \pi_{s}^{2} } \right)^{wi} } \right]^{\frac{1}{2}} } \right\} \\ \end{aligned} $$

#### Definition 7

The distance between two spherical fuzzy numbers *A* and *B* is calculated as follows:

19 $$\mathrm{dis} (\mathcal{A},\mathcal{B})=\mathrm{arccos}\left\{1-\frac{1}{2}\left({\left({\mu }_{\mathcal{A}}-{\mu }_{\mathcal{B}}\right)}^{2}+{\left({v}_{\mathcal{A}}-{v}_{\mathcal{B}}\right)}^{2}+{\left({\pi }_{\mathcal{A}}-{\pi }_{\mathcal{B}}\right)}^{2}\right)\right\} $$

This expression can also be used to calculate the spherical distance between two spherical fuzzy sets.

20 $$\mathrm{dis}\left(\mathcal{A},\mathcal{B}\right)=\frac{2}{\pi }\sum_{i=1}^{n}\mathrm{arccos}\left\{1-\frac{1}{2}\left({\left({\mu }_{\mathcal{A}}-{\mu }_{\mathcal{B}}\right)}^{2}+{\left({v}_{\mathcal{A}}-{v}_{\mathcal{B}}\right)}^{2}+{\left({\pi }_{\mathcal{A}}-{\pi }_{\mathcal{B}}\right)}^{2}\right)\right\} $$

The normalized spherical distance between *A* and *B* is defined as:

21 $${\mathrm{dis}}^{n}\left(\mathcal{A},\mathcal{B}\right)=\frac{2}{n\pi }\sum_{i=1}^{n}\mathrm{arccos}\left\{1-\frac{1}{2}\left({\left({\mu }_{\mathcal{A}}-{\mu }_{\mathcal{B}}\right)}^{2}+{\left({v}_{\mathcal{A}}-{v}_{\mathcal{B}}\right)}^{2}+{\left({\pi }_{\mathcal{A}}-{\pi }_{\mathcal{B}}\right)}^{2}\right)\right\} $$

### Spherical fuzzy SWARA

Weight assignment to criteria is one of the significant stages in MCDM problems. The SWARA method was first introduced by Keršuliene, Zavadskas [32]. In this method, a group of experts express their opinions about the options freely while a researcher takes notes and determines the relative weights by ranking them based on the evaluation of experts [5]. The SF-SWARA is applied in this research to evaluate the weights of FMEA parameters considering the probable uncertainty in the views of DMs. The SF-SWARA steps are given as follows:

*Step 1*: Sort the evaluation criteria in a descending order according to the DM’s opinions and linguistic variables (LVs).

*Step 2*: Convert the LVs expressed by DMs to SFNs using Table 1 and make a SFN decision matrix.

**Table 1:** Linguistic terms and their corresponding spherical fuzzy numbers

Linguistic variables	Spherical fuzzy number		
	*µ*	*v*	$\pi$
Absolutely More Importance (AMI)	0.90	0.10	0.1
Very High Importance (VHI)	0.80	0.20	0.2
High Importance (HI)	0.70	0.30	0.3
Slightly More Importance (SMI)	0.60	0.40	0.4
Equally Importance (EI)	0.50	0.50	0.5
Slightly Low Importance (SLI)	0.40	0.60	0.4
low Importance (LI)	0.30	0.70	0.3
Very Low Importance (VLI)	0.20	8.00	0.2
Absolutely Low Importance (ALI)	0.10	0.90	0.1

*Step 3*: Aggregate the preferences of each DM using the SWAM or SWGM operator, as shown in Eqs. (17 (18).

*Step 4*: Evaluating the comparative significance of score value:

It starts from the criteria in the second place, a score between 0 and 1 allocated by DMs to the factor *j* concerning the last criterion (*j* − 1). After applying this process to all the criteria, the comparative importance of score value (${s}_{j}$) is obtained.

*Step 5*: Compute the comparative coefficient $({k}_{j})$ as follows:

22 $$ k_{j}  = \left\{ {\begin{array}{*{20}l}    1 & {j = 1}  \\    {s_{j}  + 1} & {j > 1}  \\   \end{array} } \right.  $$

*Step 6*: Estimate the spherical fuzzy weight (${p}_{j}$) from Eq. (23).

23 $$ p_{j}  = ~\left\{ {\begin{array}{*{20}l}    1 & {j = 1}  \\    {\frac{{k_{{j - 1}} }}{{k_{j} }}} & {j > 1}  \\   \end{array} } \right. $$

*Step 7*: Estimate the relative weights of the evaluation criteria as follows:

24 $${w}_{j}= \frac{{p}_{j}}{{\sum }_{j=1}^{n}{p}_{j}}$$

where ${w}_{j}$ represents the relative weight of criterion *j* and *n* represents criteria number.

## Methodology

This study aims to propose a hybrid integrated SF-based decision-making approach for evaluating and prioritizing potential risk factors. Therefore, the proposed approach is discussed in this section.

### Problem formulation (spherical fuzzy MARCOS method)

*Step 1*: Construction of the decision matrix.

The first step in all MCDM methods aimed at prioritizing is to set up a decision matrix. In the MARCOS method, $n$ criteria evaluate $m$ alternatives, and each alternative gets a value based on each criterion. Suppose ${\mathcal{B}}_{m}=\left\{{b}_{1},{b}_{2},\ldots ,{b}_{m}\right\}$ specify our alternatives and ${\mathcal{C}}_{n}=\left\{{c}_{1},{c}_{2},\ldots ,{c}_{n}\right\}$ specify the criteria. Therefore, the decision matrix based on SF linguistic variables is first set up as an Eq. (25).

25 $${\mathfrak{K}}_{ij}={\left({C}_{j}\left({b}_{i}\right)\right)}_{m\times n} =\left(\begin{array}{lll}\left({\mu }_{11}{v}_{11}{\pi }_{11}\right)& \cdots & \left({\mu }_{1n}{v}_{1n}{\pi }_{1n}\right)\\ \vdots & \ddots & \vdots \\ \left({\mu }_{m1}{v}_{m1}{\pi }_{m1}\right)& \cdots & \left({\mu }_{mn}{v}_{mn}{\pi }_{mn}\right)\end{array}\right)$$

*Step 2*: Conversion of linguistic variables to SF numbers.

The decision matrix formed in the first step is converted to SFNs utilizing Table 1.

*Step 3*: Determining the ideal and the counter-ideal.

In this step, ideal ($\mathop{\text{A}}\limits^{\circ}_{ai}$) and anti-ideal ($\mathop{\text{A}}\limits^{\circ}_{id}$) values are find out based on Eqs. (26) and (27),

26 $$ \begin{array}{*{20}c} {\mathop{\text{A}}\limits^{\circ}_{ai} = \mathop {\min }\limits_{1 \le i \le m} x_{ij} . j\, \in \,B^{\max } , } & { \mathop {\min }\limits_{1 \le i \le m} x_{ij} . j\, \in \,C^{\min } } \\ \end{array} $$

27 $$ \begin{array}{*{20}c} {\mathop{\text{A}}\limits^{\circ}_{id} = \mathop {\max }\limits_{1 \le i \le m} x_{ij} . j\, \in \,B^{\max } , } & {\mathop {\min }\limits_{1 \le i \le m} x_{ij} . j\, \in \,C^{\min } } \\ \end{array} $$

where *B* signifies the benefit aspect criteria, and *C* signifies the criteria with a cost aspect.

*Step 4*: Weighing decision matrix.

Form the weighted decision matrix ${v}_{ij}$ by multiplying the elements of matrix ${\mathfrak{K}}_{ij}$ with corresponding criteria weight coefficients based on Eq. (29).

28 $${v}_{ij}={w}_{j}.{x}_{ij}=({\mu }_{ij}^{v}.{v}_{ij}^{v}.{\pi }_{ij}^{v})$$

29 $${w}_{j}.{x}_{ij}=\left\{{\mu }_{j}^{w}.{\mu }_{ij}^{x}{\left[{\left({v}_{j}^{w}\right)}^{2}+{\left({v}_{ij}^{x}\right)}^{2}-{\left({v}_{j}^{w},{v}_{ij}^{x}\right)}^{2}\right]}^{1/2}.{\left[\left(1-{\left({v}_{j}^{w}\right)}^{2}\right).{\left({\pi }_{ij}^{x}\right)}^{2}+\left(1-{\left({v}_{ij}^{x}\right)}^{2}\right).{\left({\pi }_{j}^{w}\right)}^{2}-{({\pi }_{j}^{w}.{\pi }_{ij}^{x})}^{2}\right]}^{1/2}\right\}$$

*Step 5*: Determine the utility degrees (UG) of alternatives.

In this step, the UGs of alternatives are calculated utilizing Eqs. (30) and (31).

30 $${K}_{i}^{+}=\frac{2}{n.\pi }\sum_{j=1}^{n}\mathrm{arccos}({\mu }_{ij}^{v}.{\mu }_{id j}^{v}+{v}_{ij}^{v}.{v}_{id j}^{v}+{\pi }_{ij}^{v}.{\pi }_{id j}^{v})$$

31 $${K}_{i}^{-}=\frac{2}{n.\pi }\sum_{j=1}^{n}\mathrm{arccos}({\mu }_{ij}^{v}.{\mu }_{ai j}^{v}+{v}_{ij}^{v}.{v}_{ai j}^{v}+{\pi }_{ij}^{v}.{\pi }_{ai j}^{v})$$

*Step 6*: Determine the utility functions of each alternative and ranking.

Finally, the utility functions of each alternative are calculated using Eq. (32).

32 $${F}_{i}=\frac{{\mathcal{K}}_{i}^{+}+{\mathcal{K}}_{i}^{-}}{1+\frac{1-f\left({\mathcal{K}}_{i}^{+}\right)}{f\left({\mathcal{K}}_{i}^{+}\right)}+\frac{1-f\left({\mathcal{K}}_{i}^{-}\right)}{f\left({\mathcal{K}}_{i}^{-}\right)}}$$

where $f\left({\mathcal{K}}_{i}^{-}\right)$ and $f\left({\mathcal{K}}_{i}^{+}\right)$ represent the utility functions of alternatives, calculated according to:

33 $$f\left({\mathcal{K}}_{i}^{-}\right)=\frac{{\mathcal{K}}_{i}^{+}}{{\mathcal{K}}_{i}^{+}+{\mathcal{K}}_{i}^{-}}$$

34 $$f\left({\mathcal{K}}_{i}^{+}\right)=\frac{{\mathcal{K}}_{i}^{-}}{{\mathcal{K}}_{i}^{+}+{\mathcal{K}}_{i}^{-}}$$

*Step 7*: Determine the ranks of the alternative.

Rank the alternatives according to the parameter ${F}_{i}$ in descending order.

### Proposed method

**Fig. 1:**
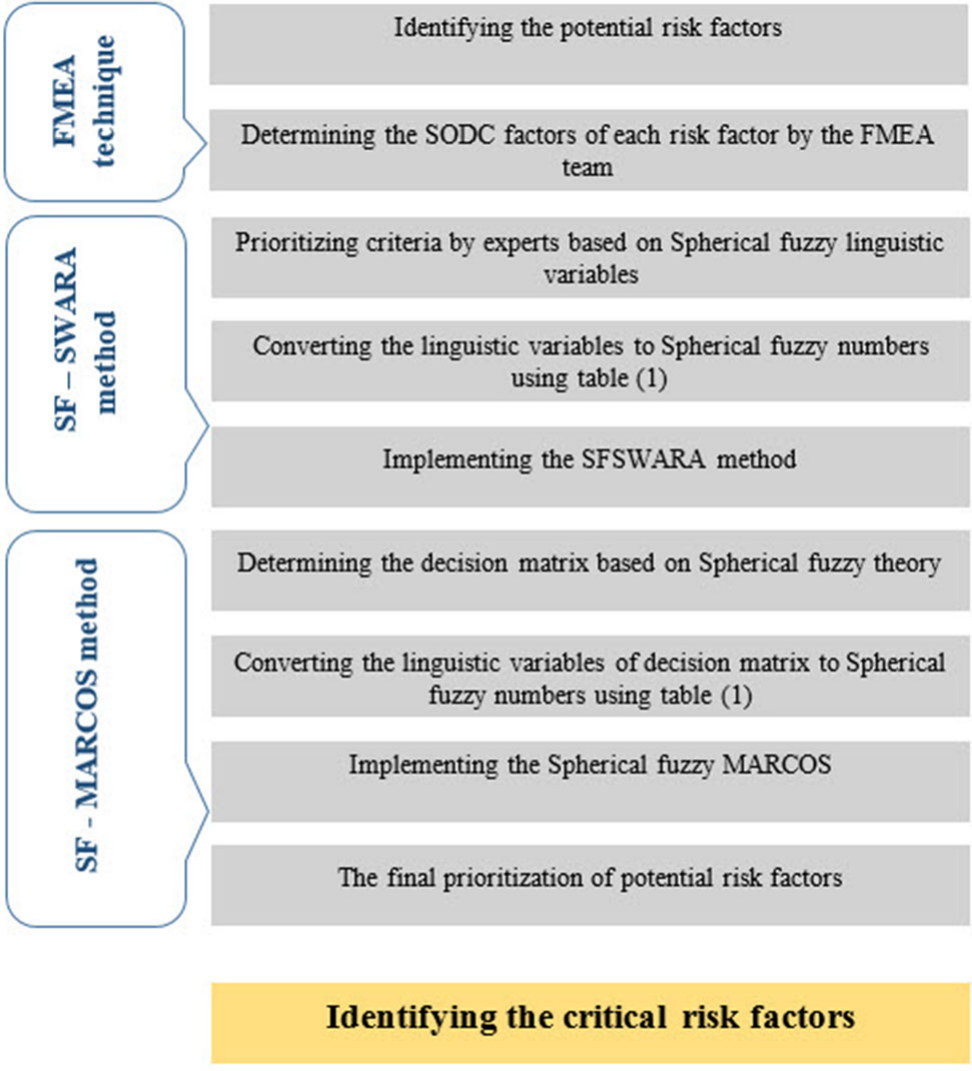
The flow of the proposed approach

In this section, a systematic approach of extended SWARA and MARCOS methods is proposed to assess the potential risk factors. The proposed approach is presented in three steps as follows:

According to Fig. 1, in phase one, the FMEA team identifies potential risk factors, and a value is assigned to each of the four criteria described in the FMEA process (severity, occurrence, detection, and cost). The FMEA team then uses Table 1 and linguistic variables (LVs) to figure out the values of SODC factors for each identified risk factor. So that the lowest and highest scores of SODC factors for each potential risk factor are determined by LVs of Absolutely Low Importance (ALI) and Absolutely More Importance (AMI), respectively. Also, the significance values are shown using a 9-point rating scale from ALI to AMI.

Then, in the second phase, the importance of the criteria (severity, occurrence, detection, and cost) is determined using spherical fuzzy LVs by the experts' team, which consists of 3 experts with experience and expertise in various fields. Using Table 1, the LVs are turned into spherical fuzzy numbers. Then, the preferences of the experts are combined, and the SFSWARA method is used to get the final weights of the SODC factors as a spherical fuzzy number.

In the third phase, based on the outputs obtained in the first and second phases, an attempt is made to prioritize the FMEA team’s identified potential risk factors based on the weights of the criteria. In the SF-MARCOS method, after figuring out the decision matrix (the result of the first step), which is made up of spherical fuzzy LVs, Table 1 is used to turn these values into spherical fuzzy numbers. Then, a weighted spherical fuzzy decision matrix is organized using weights of SODC factors (output of the second phase). The SF-MARCOS method is utilized to prioritize the potential risk factors in the following. In prioritizing the potential risk factors based on the score obtained from the proposed approach, potential risk factors with a higher score will be ranked as the first priority. The flowchart in Fig. 1 shows the proposed approach to rank the potential risk factors.

## Results and discussion

In recent years, in line with a global goal to reduce road accident casualties, more extensive research and studies have been conducted to increase the level of road safety [69–76]. This target is one of the United Nations Sustainable Development Goals (SDGs) and is connected to wellness and urban living. Extensive and valuable studies have been conducted on road safety and the parameters involved in causing accidents. However, due to the uncertainty in examining these factors, the need for further studies is fully felt. Therefore, considering the roads of the Calabria region in southern Italy as the case study in this research, the methodology proposed in the previous sections is applied to this case. More discussion regarding the risks of the case study will be given in the following section.

### Context definition

**Fig. 2:**
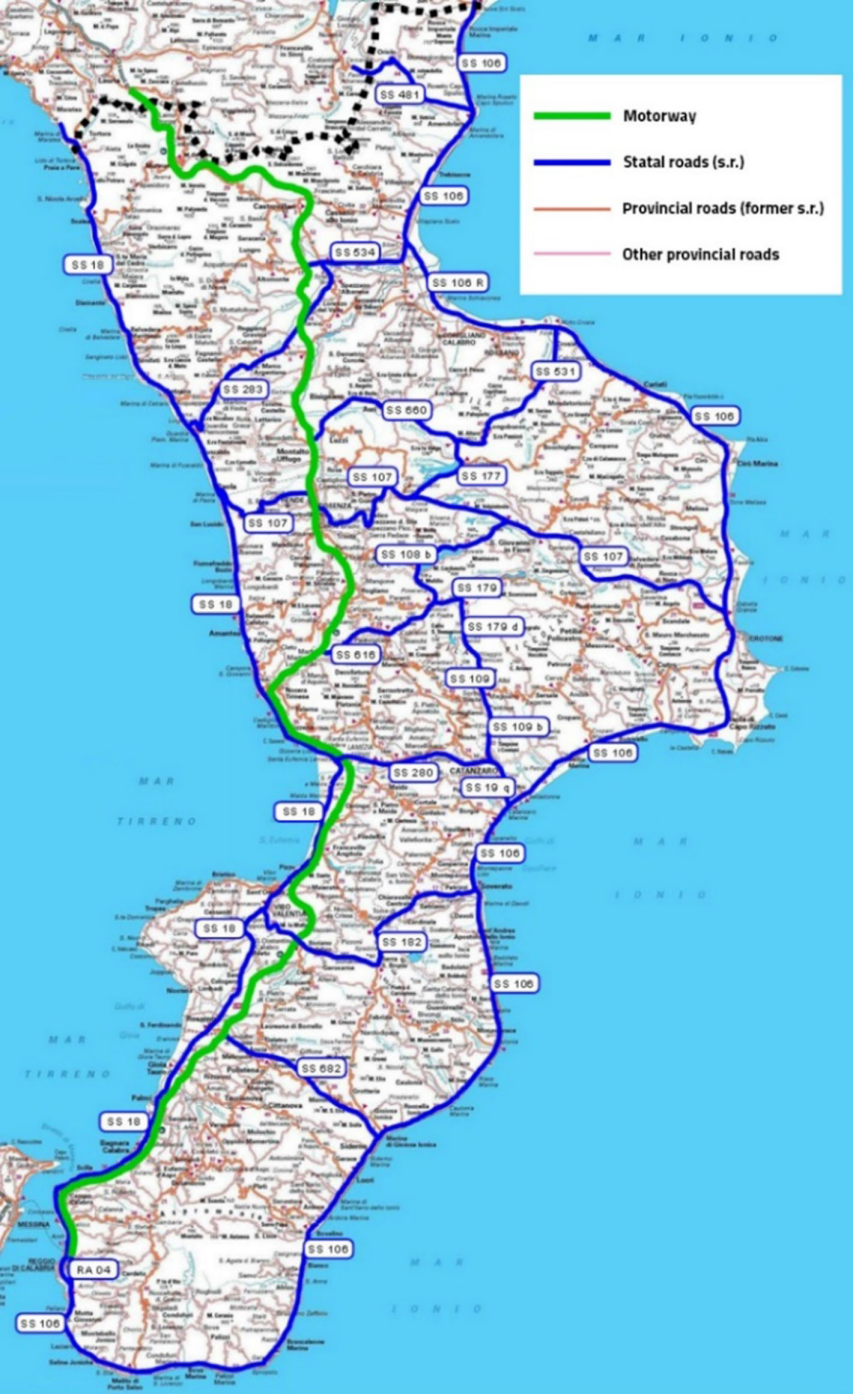
The Calabrian rural road network (color figure online)

The approach introduced in the paper is applied to a study context that coincides with the rural road network of the Calabria region. Based on the Piano Regionale dei Trasporti [77], the Calabrian rural road network has an extension of 9066 km, divided as follows (Fig. 2):

- 294 km of motorway, forming the Calabrian section of the A2 “Salerno-Reggio Calabria” motorway,
- 5.6 km of motorway links,
- 1321.5 km of statal roads,
- 19.1 km of extra-urban roads under classification or declassification,
- 7426 km of provincial roads.

From an administrative point of view, except for the provincial roads, the other Calabrian rural roads (1640 km) are managed by ANAS, the concessionaire company for managing the road and motorway network of national interest in Calabria.

The A2 Salerno-Reggio Calabria motorway, currently being modernized, is the main road in Calabria; it ensures the region's connections with Italy (and more generally with Europe), absorbs traffic flows in transit through Sicily, and guarantees long-distance connections within the regional territory.

The state roads represent the main axes of the regional road network of national interest and are required to ensure rapid inter-provincial or intra-provincial connections.

The provincial roads constitute the preponderant component of the Calabrian road infrastructure patrimony; they integrate the network constituted by the motorway and the state roads, ensuring the connection of the municipal areas to them.

According to ISTAT [78], in Calabria, from 2001 to 2010 road fatalities fell by 20.2%, less than the national average (− 42.0%). Between 2010 and 2020, there were − 55.8% and − 41.8% variations. In the same period, the mortality rate decreased from 4.1 to 2.9 deaths per 100 accidents, while the national average recorded a slight increase (1.9 to 2.0 deaths per 100 accidents).

In 2020, the most significant number of accidents occurred on urban roads (1286, 61.8% of the total), causing 26 dead (42.6% of the total) and 1904 injured (58.3%). The most serious accidents occur on other roads (4.8 deaths per 100 accidents) and motorways (2.8 deaths per 100 accidents).

Despite the indexes of injury, mortality, and severity decreased in the 2 years 2019–2020^1^ (respectively from 164.2 to 157.0, from 3.8 to 2.9, and from 2.2 to 1.8), the accident rate remains high on the A2 Salerno-Reggio Calabria motorway (117 accidents, 4 deaths, and 177 injured in 2020) and along the two main coastal roads, especially for the Ionic SS106 state road, along which the highest number of accidents is recorded (200 accidents, 9 deaths, and 335 injuries), and of the Tyrrhenian SS18 state road. In inland areas, the accident rate remains high along the SS280 state road called “dei Due Mari,” the SS283 state road called “delle Terme Luigiane” and the SS682 state road called “del Passo della Limina.”

Under these assumptions, intending to assess the risk of an accident and the related components, following the approach introduced in the previous sections, the team of experts identified a set of serious potential risk factors in the rural roads of Calabria above described (Table 2). In fact, this team of experts, in the form of a research group, by identifying and collecting risks that are generally considered in road safety, considered more than 25 risks in the first stage. Then, in several meetings of these experts, among these collected risks, 16 risks were selected as potential risks that could be taken in this case study. It is important to note that the goals of achieving sustainable mobility were taken into account when these risks were first looked at and chosen.

**Table 2:** A set of serious potential risk factors in rural roads of Calabria

Number of risks	Title of risk	Risk sources
R.F. 1	Age of driver	Driver conditions
R.F. 2	Presence of alcohol, medicinal or recreational drugs	
R.F. 3	Fatigue	
R.F. 4	Talk on the phone	
R.F. 5	Overtaking	
R.F. 6	Lack of attention to the longitudinal safety distance	
R.F. 7	Lack of driving skills	
R.F. 8	Handling of vehicle	Vehicle condition
R.F. 9	Age of vehicle	
R.F. 10	Whether condition	Environmental conditions
R.F. 11	Road design Mistakes	Engineering and technically
R.F. 12	Defect of maintenance	
R.F. 13	Lack of lighting	
R.F. 14	Inadequate penalties	Policies
R.F. 15	Landslides	Unpredictable Risks
R.F. 16	Floods	

### Results

In this section, the results of the proposed approach in assessing serious potential risk factors in rural roads of Calabria are examined.

This study aims to introduce a new approach using MCDM methods in an environment of uncertainty. According to the first step of the proposed approach, experts first express the importance of each criterion according to Table 3 in linguistic terms using Table 1.

**Table 3:** The importance of criteria in the form of SF linguistic variables

Criteria	DM1	DM2	DM3
Severity	HI	VHI	HI
Occurrence	SLI	LI	SLI
Detection	LI	VLI	VLI
Cost	HI	SMI	SMI

The LVs assigned by the experts are then transformed to SFNs according to Table 1. Utilizing Eqs. (17) and (18) and the experts’ weights, which are 0.2, 0.3, and 0.5, respectively, the aggregated decision matrix is created (see Table 4).

**Table 4:** Aggregated SF decision matrix based on SWAM

SWAM			
Criteria	Weights of criteria		
	*µ*	*v*	$\pi$
S	0.724	0.276	0.301
O	0.382	0.618	0.400
D	0.235	0.768	0.256
C	0.634	0.366	0.352

The SVs of SFNs are obtained using Eq. (14), and the matrix S is formed in the next step. Then, according to the score function value, the criteria are sorted in descending order, and ${k}_{j}$ and ${p}_{j}$ are calculated utilizing Eqs. (22) and (23). Finally, ${w}_{j}$, which is the final weight of the criteria, is calculated by Eq. (24) (Table 5).

**Table 5:** The results obtained from the SF-SWARA method

Criteria	Score values	$S_{j}$	$K_{j}$	$P_{j}$	$W_{j}$
S	0.177	–	1	1	0.295
C	0.079	0.098	1.098	0.910	0.269
O	− 0.047	0.126	1.126	0.808	0.238
D	− 0.261	0.214	1.214	0.665	0.196

The SF-MARCOS method is then applied to prioritize risk factors. According to the first step of this method, the decision matrix is formed by the FMEA team with SF linguistic terms (Table 6). The FMEA team have been constructed by selecting some experts in the safety and risk management field and some specialists from traffic department.

**Table 6:** Evaluation of failure states based on SF linguistic variables

Risk factors	Severity	Occurrence	Detection	Cost
R.F. 1	EI	HI	SLI	SMI
R.F. 2	VHI	SMI	SMI	SMI
R.F. 3	SMI	SLI	SMI	HI
R.F. 4	HI	SMI	EI	HI
R.F. 5	EI	HI	EI	HI
R.F. 6	SMI	HI	EI	VHI
R.F. 7	VHI	EI	HI	SMI
R.F. 8	EI	SMI	SLI	EI
R.F. 9	EI	SMI	EI	SMI
R.F. 10	SMI	SMI	EI	SMI
R.F. 11	VLI	SLI	SLI	EI
R.F. 12	SMI	HI	SLI	SMI
R.F. 13	EI	HI	LI	EI
R.F. 14	VLI	SMI	EI	HI
R.F. 15	SLI	EI	SLI	EI
R.F. 16	SLI	SLI	SLI	SLI

Then, decision matrices based on SF variables are transformed to SFNs utilizing Table 1. The weights obtained from the SWARA method are applied (see Table 7).

**Table 7:** The weighted SF decision matrix

Risk factors	Severity			Occurrence			Detection			Cost		
	*µ*	*v*	$\pi$	*µ*	*v*	$\pi$	*µ*	*v*	$\pi$	*µ*	*v*	$\pi$
R.F. 1	0.285	0.815	0.322	0.385	0.750	0.196	0.184	0.904	0.198	0.336	0.781	0.257
R.F. 2	0.510	0.622	0.159	0.318	0.803	0.244	0.290	0.835	0.224	0.336	0.781	0.257
R.F. 3	0.351	0.763	0.267	0.202	0.885	0.217	0.290	0.835	0.224	0.407	0.723	0.206
R.F. 4	0.425	0.701	0.214	0.318	0.803	0.244	0.235	0.873	0.269	0.407	0.723	0.206
R.F. 5	0.285	0.815	0.322	0.385	0.750	0.196	0.235	0.873	0.269	0.407	0.723	0.206
R.F. 6	0.351	0.763	0.267	0.385	0.750	0.196	0.235	0.873	0.269	0.490	0.649	0.154
R.F. 7	0.510	0.622	0.159	0.258	0.847	0.294	0.352	0.789	0.181	0.336	0.781	0.257
R.F. 8	0.285	0.815	0.322	0.318	0.803	0.244	0.184	0.904	0.198	0.273	0.830	0.309
R.F. 9	0.285	0.815	0.322	0.318	0.803	0.244	0.235	0.873	0.269	0.336	0.781	0.257
R.F. 10	0.351	0.763	0.267	0.318	0.803	0.244	0.235	0.873	0.269	0.336	0.781	0.257
R.F. 11	0.109	0.936	0.111	0.202	0.885	0.217	0.184	0.904	0.198	0.273	0.830	0.309
R.F. 12	0.351	0.763	0.267	0.385	0.750	0.196	0.184	0.904	0.198	0.336	0.781	0.257
R.F. 13	0.285	0.815	0.322	0.385	0.750	0.196	0.136	0.932	0.141	0.273	0.830	0.309
R.F. 14	0.109	0.936	0.111	0.318	0.803	0.244	0.235	0.873	0.269	0.407	0.723	0.206
R.F. 15	0.224	0.860	0.240	0.258	0.847	0.294	0.184	0.904	0.198	0.273	0.830	0.309
R.F. 16	0.224	0.860	0.240	0.202	0.885	0.217	0.184	0.904	0.198	0.214	0.872	0.230
ai	0.109	0.763	0.228	0.202	0.847	0.279	0.352	0.873	0.330	0.214	0.781	0.230
id	0.510	0.763	0.343	0.385	0.681	0.128	0.136	0.904	0.245	0.490	0.723	0.238

Weighted decision matrix is calculated using Eq. (5), which is as follows for the first risk factor and severity criterion:

$${W}_{s}*\left({\mu }_{R.F.1,s},{v}_{R.F.1,s},{\pi }_{R.F.1,s}\right)=\left[\sqrt{1-{\left(1-{0.5}^{2}\right)}^{0.295} }.{0.5}^{0.295}.\sqrt{{\left(1-{0.5}^{2}\right)}^{0.295}-{\left(1-{0.5}^{2}-{0.5}^{2}\right)}^{0.295}}\right]=(0.285, 0.815, 0.322)$$

Also, for the first risk factor and occurrence criterion:

$${W}_{o}*\left({\mu }_{R.F.1,o},{v}_{R.F.1,o},{\pi }_{R.F.1,o}\right)=\left[\sqrt{1-{\left(1-{0.7}^{2}\right)}^{0.238} }.{0.3}^{0.238}.\sqrt{{\left(1-{0.7}^{2}\right)}^{0.238}-{\left(1-{0.7}^{2}-{0.3}^{2}\right)}^{0.238}}\right]=(0.385, 0.750, 0.196)$$

According to the next step, the utility degrees $$({K}_{i}^{+},{K}_{i}^{-} )$$, the utility functions of alternatives$$(f{(K}_{i}^{+}), f{(K}_{i}^{-}))$$, and the final scores of alternatives ($${F}_{i})$$ are calculated using Eqs. (30–34), respectively, for each risk factor (Table 8).

**Table 8:** The results from the MARCOS method

Risk factors	$K_{i}^{ + }$	$K_{i}^{ - }$	$f(K_{i}^{ + } )$	$f(K_{i}^{ - } )$	$F_{i}$	Rank
R.F. 1	0.389	0.413	0.514	0.485	0.267	9
R.F. 2	0.425	0.440	0.508	0.491	0.288	2
R.F. 3	0.410	0.415	0.503	0.496	0.275	5
R.F. 4	0.408	0.426	0.510	0.489	0.277	4
R.F. 5	0.396	0.417	0.512	0.487	0.270	7
R.F. 6	0.410	0.438	0.516	0.483	0.282	3
R.F. 7	0.434	0.440	0.503	0.496	0.291	1
R.F. 8	0.381	0.392	0.507	0.492	0.257	14
R.F. 9	0.387	0.395	0.505	0.494	0.260	12
R.F. 10	0.395	0.406	0.506	0.493	0.266	10
R.F. 11	0.400	0.375	0.483	0.516	0.258	13
R.F. 12	0.397	0.423	0.515	0.484	0.272	6
R.F. 13	0.383	0.408	0.515	0.484	0.263	11
R.F. 14	0.413	0.399	0.491	0.508	0.270	8
R.F. 15	0.386	0.379	0.495	0.504	0.255	16
R.F. 16	0.392	0.379	0.491	0.508	0.256	15

For example, ${{K}}_{{i}}^{+}$ and ${{K}}_{{i}}^{-}$ for the first risk factor are calculated using Eqs. (30, 31) as follows:

$${K}_{1}^{+}=\frac{2}{4\times 3.14}\sum_{j=1}^{4}\mathrm{arccos}(0.285\times 0.510+0.815\times 0.763+\dots +0.257\times 0.238)=0.38924$$

$${K}_{1}^{-}=\frac{2}{4\times 3.14}\sum_{j=1}^{4}\mathrm{arccos}(0.285\times 0.109+0.815\times 0.763+\dots +0.257\times 0.230)=0.41284$$

In the next step, $f\left({\mathcal{K}}_{1}^{+}\right)$ and $f\left({\mathcal{K}}_{1}^{-}\right)$ values which present utility functions of first risk factor, calculated using Eqs. (34, 33), respectively.

$$f\left({\mathcal{K}}_{1}^{-}\right)=\frac{0.389}{0.389+0.413}=0.48529$$

$$f\left({\mathcal{K}}_{1}^{+}\right)=\frac{0.413}{0.389+0.413}=0.51471$$

At the end, the final score of the first risk factor (${F}_{i}$), calculated using Eq. (32).

$${F}_{1}=\frac{0.389+0.413}{1+\frac{1-0.514}{0.514}+\frac{1-0.485}{0.485}}=0.26705$$

According to Table 8, we find that R.F. 7, with a score of 0.291, has a higher priority than other risk factors. R.F. 2 and R.F. 6, with 0.288 and 0.282 scores, are in the second and third priority, and R.F. 15, with score 0.255, is in the last priority. Therefore, based on this prioritization, specialists can take preventive and corrective measures to avoid the negative effects of these potential risk factors. In fact, this ranking, which is based on the opinions of experts, can give a comprehensive picture of all the possible risks that have been looked into for this case study, so that preventive measures can be taken and the situation can be made better. This ranking was independent of the classification of risks based on controlled or uncontrolled risks and was done only based on the experience and expertise of experts who were fully familiar with the conditions of the roads and the environment of the case study.

### Validation of results

**Fig. 3:**
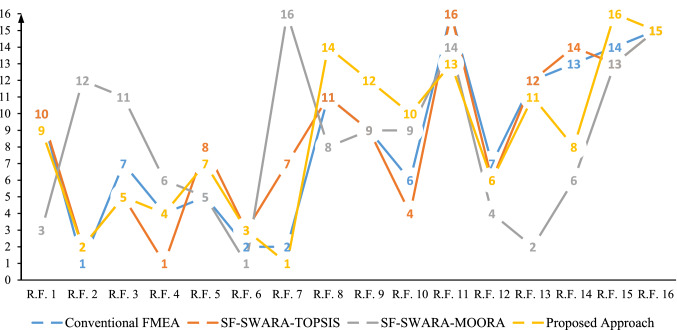
Comparison of prioritization of the Risk Factors with various approaches (color figure online)

This section provides some validation tests of the obtained rankings. For the first validation study, a comparative study between the proposed approach's results (modified FMEA by SF-SWARA-MARCOS) and some existing approaches include SF-TOPSIS, SF-MOORA, and conventional FMEA (RPN score) has been performed. According to Table 9, based on conventional FMEA (RPN score), risk factor 2 (R.F. 2) has a higher priority than other risk factors. In addition, R.F. 6 and R.F. 7 are both in second priority. By examining the priority of potential risk factors based on the RPN score, it can be concluded that the ranking of potential risk factors is done so that the potential risk factors are placed in 14 categories instead of 16 categories. This issue indicates that rank based on the conventional index is not entirely done and perplexes decision-maker in risk management and planning corrective/preventive estimates. In line with the conventional FMEA approach results shown in Table 9, incomplete rank can be because of the lack of allocation of various weights (according to experts) to SODC factors and lack of uncertainty in the values of these factors. The problem of duplicate prioritizes has been solved by applying the SF-SWARA-TOPSIS method and risk factors are placed in 16 categories. Base on the results of applying this method, R.F.4 has the highest priority, and R.F. 2 and R.F. 6 are in the second and third priority, respectively. By applying SF-SWARA-MOORA method, the problem of duplicate priorities has been appeared again where R.F. 4 and R.F. 14 are both in sixth priority. Also, both of R.F. 9 and R.F. 10 have been ranked eight. On the other hand, according to the prioritization resulting from the proposed approach, the R.F. 7 and R.F. 2 are critical risk factors, respectively (see Fig. 3). Then, by examining the table further, it is observed that when applying SF-SWARA-MARCOS approach, the defect of the incomplete rank problem of risk factors is eliminated; the results of this method are closer to reality by applying uncertainty in the expert’s opinions. It has been demonstrated that spherical fuzzy sets serve as the theoretical basis for MCDM's greater capability in dealing with ambiguous problems. Thus, more than classical fuzzy sets, it reflects uncertainty in real-world issues, which leads to the project's critical path being determined correctly, and as a result, project planning is closer to reality.

**Table 9:** The results from the MARCOS method

Risk factors	Conventional FMEA		SF-SWARA-TOPSIS		SF-SWARA-MOORA		Proposed Approach	
	RPN	Rank	Closeness Ratio	Rank	*Ny*_*i*_i	Rank	*F*_*i*_	Rank
R.F. 1	840	8	− 0.258	10	10.860	3	0.267	9
R.F. 2	1728	1	− 0.092	2	6.389	10	0.288	2
R.F. 3	1008	6	− 0.160	5	6.521	9	0.275	5
R.F. 4	1470	3	− 0.043	1	8.919	6	0.278	4
R.F. 5	1225	4	− 0.193	8	10.076	5	0.271	7
R.F. 6	1680	2	− 0.095	3	11.751	1	0.282	3
R.F. 7	1680	2	− 0.183	7	4.396	14	0.292	1
R.F. 8	600	9	− 0.346	11	7.519	7	0.258	14
R.F. 9	900	7	− 0.244	9	7.456	8	0.261	12
R.F. 10	1080	5	− 0.147	4	7.456	8	0.267	10
R.F. 11	160	14	− 0.697	16	5.578	12	0.258	13
R.F. 12	1008	6	− 0.163	6	10.179	4	0.273	6
R.F. 13	525	10	− 0.387	12	11.637	2	0.264	11
R.F. 14	420	11	− 0.468	14	8.919	6	0.271	8
R.F. 15	400	12	− 0.454	13	6.254	11	0.255	16
R.F. 16	256	13	− 0.508	15	4.716	13	0.257	15

**Fig. 4:**
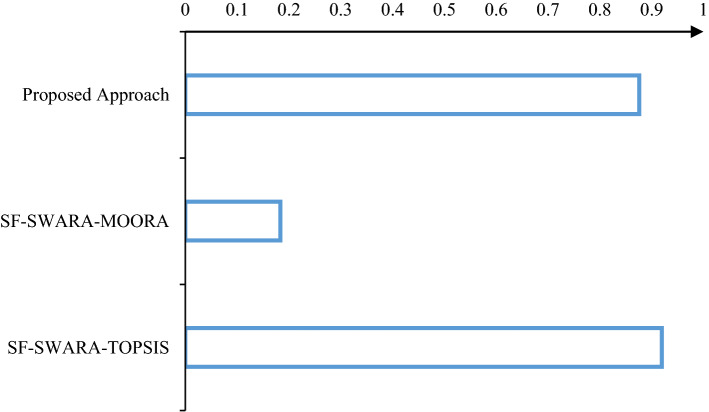
Correlation plot of the proposed approach and existing approaches with classic FMEA

By comparing the results obtained by applied approaches, it can be observed from Fig. 4 that SF-MOORA method results have high variations compared to other investigated methods. It seems that this method is not suitable to solve the problem of this study with correlation coefficient as 0.18334. On the other hand, when we compare the results obtained by SF-SWARA-TOPSIS and proposed approach (SF-SWARA-MARCOS), we observe that there are few rank variations between each of them with conventional FMEA. The correlation coefficient is observed as 0.87656 for proposed approach and 0.91982 for SF-SWARA-TOPSIS. It can be obtained from this results that this proposed approach is valid because the detected results of this approach is similar to the results of SF-TOPSIS approach as a reliable method in literature [61, 79]. Although the coefficient of SF-SWARA-TOPSIS is higher than the proposed approach of this study, in the detailed examination, the result of this examination is different. The critical risk factors that obtained from conventional FMEA are more close to critical risk factors that obtained from the proposed approach from SF-SWARA-TOPSIS, where R.F. 7 is in the first and second priority (Table 9) in the results of the conventional FMEA and SF-SWARA-MARCOS, but it is in the seventh priority according to SF-SWARA-TOPSIS approach. Although we do detect extreme rank variations between the proposed approach in this study and some existing approaches, it can be declared that the application of this proposed approach is new in the FMEA domain.

A sensitivity analysis was done as a second validation study. Here, the variation in risk factor ranking is analyzed with regard to changes in criterion weights. We exchange the weight values of the modified FMEA parameters for the sensitivity analysis. As there are four risk parameters in the modified FMEA, in our case study, eight combinations (current scenario and seven different scenarios) are created.

The ranking orders of 16 risk factors with reference to the eight weight values are shown in Table 10. It can be observed from Table 10 that when the weight value varies, there are variations in the ranking orders of risks. As a result, our method is sensitive to the weights of the modified FMEA risk parameters. While R.F. 7 and R.F. 2 are ranked as the most crucial risk factors, the least critical risk factor varies depending on the weight values. Based on the comparative evaluation of the results obtained from our proposed approach with related literature studies [61, 80, 81], we can state that the ranking result obtained by our approach is credible and valid.

**Table 10:** Rank changes depending on the criteria’s weight changes

Risk factors	Current weight		Sc. 1		Sc. 2		Sc. 3		Sc. 4		Sc. 5		Sc. 6		Sc. 7	
	$F_{i}$	Rank	$F_{i}$	Rank	$F_{i}$	Rank	$F_{i}$	Rank	$F_{i}$	Rank	$F_{i}$	Rank	$F_{i}$	Rank	$F_{i}$	Rank
R.F. 1	0.267	9	0.259	10	0.252	10	0.239	9	0.252	9	0.257	8	0.224	7	0.216	10
R.F. 2	0.288	2	0.281	2	0.273	2	0.257	2	0.273	2	0.274	2	0.228	3	0.231	2
R.F. 3	0.275	5	0.268	5	0.261	6	0.245	6	0.260	5	0.263	5	0.224	5	0.221	8
R.F. 4	0.278	4	0.271	4	0.263	4	0.248	5	0.262	4	0.265	4	0.221	9	0.223	5
R.F. 5	0.271	7	0.263	8	0.256	8	0.242	8	0.254	8	0.260	7	0.223	8	0.218	9
R.F. 6	0.282	3	0.274	3	0.267	3	0.251	3	0.265	3	0.270	3	0.231	2	0.226	4
R.F. 7	0.292	1	0.285	1	0.277	1	0.260	1	0.277	1	0.277	1	0.232	1	0.233	1
R.F. 8	0.258	14	0.250	15	0.244	15	0.231	16	0.244	14	0.248	13	0.214	12	0.209	16
R.F. 9	0.261	12	0.253	12	0.247	13	0.233	13	0.246	13	0.251	12	0.213	13	0.211	15
R.F. 10	0.267	10	0.259	9	0.253	9	0.239	10	0.252	10	0.255	10	0.215	11	0.216	11
R.F. 11	0.258	13	0.253	13	0.249	11	0.239	11	0.247	12	0.245	15	0.209	15	0.221	7
R.F. 12	0.273	6	0.265	7	0.258	7	0.244	7	0.258	6	0.262	6	0.225	4	0.221	6
R.F. 13	0.264	11	0.256	11	0.249	12	0.236	12	0.250	11	0.254	11	0.224	6	0.214	12
R.F. 14	0.271	8	0.266	6	0.261	5	0.249	4	0.257	7	0.257	9	0.218	10	0.229	3
R.F. 15	0.255	16	0.249	16	0.244	16	0.232	15	0.243	16	0.244	16	0.209	16	0.212	13
R.F. 16	0.257	15	0.251	14	0.244	14	0.232	14	0.243	15	0.247	14	0.210	14	0.211	14

**Fig. 5:**
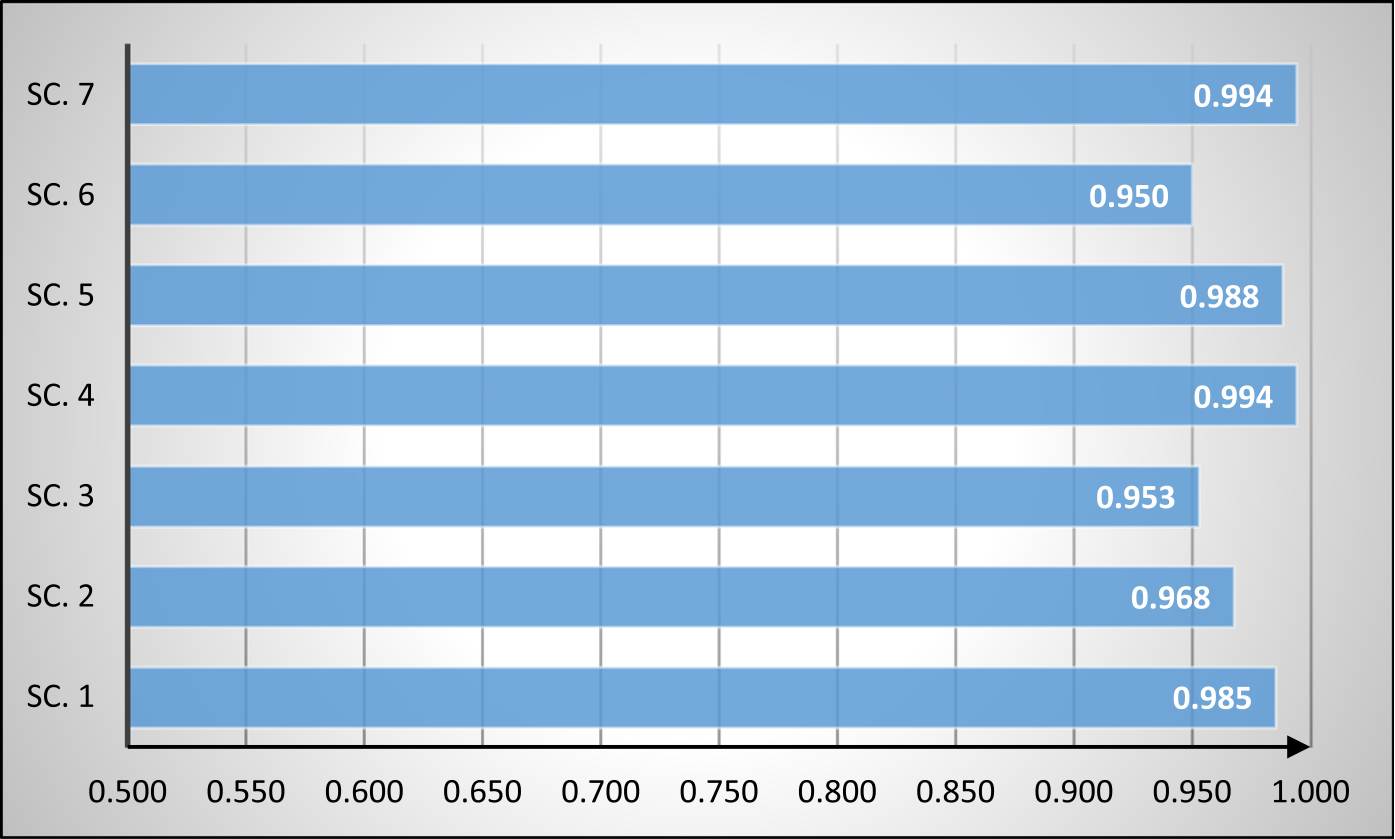
Comparison of correlations between scenario results and main ranking orders

The correlation between the scenario results and the main ranking orders with regard to weight values is also examined. According to Fig. 5, SC.7 and SC.4 has highest correlation with the main current scenario results with 0.994 coefficient. Also, it can observed that SC. 6 has the least correlation with the main current scenario results with 0.950 coefficient. As a result of this analysis, it is clearly visible that ranking results based on the seven different weight values have a very high correlation.

Figure 5 displays a correlation coefficient more significant than 95% for all the results that show a high and positive correlation.

As previously stated, the risk assessment process in road safety is an essential step in achieving sustainable mobility. Nevertheless, uncertainty is the most undesirable effect affecting the risk assessment process. So, an integrated approach in a spherical fuzzy environment for accurate assessment of risks can be helpful to minimize the uncertainty effects of the risk assessment process. The present study aims to present an integrated approach for risk assessment and prioritizing rural road safety in Calabria, in southern Italy. For this aim, the developed approach of FMEA with the SF-SWARA-MARCOS method was presented in this study. In this regard, numerous risk factors were identified and then consulted with transportation and road safety experts who were familiar with the study area. Then, sixteen risk factors were selected from six risk sources, including driver conditions, vehicle conditions, environmental conditions, engineering and technological conditions, policies, and unpredictable risks. Finally, the developed approach was applied to assess and rank the sixteen risk factors. The following is an explanation of the study's findings.

According to the proposed approach, the first source of risk affecting rural road safety in Calabria is the driver’s condition. As many as five risk factors associated with driver conditions are at the top of the ranking (Lack of driving skills, Presence of alcohol, medicinal or recreational drugs, Lack of attention to longitudinal safety distance, Talk on the phone, and Fatigue, respectively). These findings are in line with the evidence of scientific research and with the data collected on a global scale, which highlight that human factors play a very significant role in sustainable transportation systems, causing almost 90% of all road accidents [82–85].

In order to reduce the risks caused by drivers, effective measures should be taken to improve and control the behavior of drivers themselves. At present, the common measures are mainly financial penalties. These measures can include periodic training, control, and monitoring of drivers' behavior at regular or unexpected times. Also, drivers can get training and rules-related reminders all the time by using modern technologies like smartphones, text messages, and training applications. Furthermore, enacting and enforcing legislation on key risk factors could be advisable. Also, it will be important to come up with ways to measure some key performance indicators (KPIs) set by Agenda 2030. These KPIs include speed, the use of protection systems (helmets, seat belts, and child seats), the use of alcohol and drugs, the safety level of the vehicle fleet and the national road network, distractions while driving, and how well rescue systems work in case of an accident.

The last two risk factors had the lowest level of risk (unpredictable risks). The ranking results of these two risks were in full accordance with the conditions prevailing on the roads.

It is also suggested that for further studies, the methodology used in this study should not only be used for other rural roads in Italy and their results should be compared with each other, but it is also suggested to be used on urban roads as well, so that by examining urban road risks, we can use this method to check the safety of roads in urban areas in fuzzy environments and under uncertainty. Although the results are generally in line with national data, it should be emphasized that they are specific, especially concerning the individual factors analyzed; they should only be utilized on rural roads in the Calabria region and not on other rural roads. A more detailed analysis could be carried out by examining other factors that have a more significant impact on the risk of a road accident in urban areas.

## Conclusions

This study presented the developed approach of FMEA with SF-SWARA and SF-MARCOS methods. Each method was utilized to cover several shortcomings of the traditional FMEA method in order that after determining the probable flaw scenarios are based on FMEA, SF-SWARA is used to count the weight of factors, and SF-MARCOS is utilized to prioritize potential risk factors. Actually, an integrated SF context decision framework is provided in the weighting phase and the prioritization phase. It was the first time that integrated SF-SWARA-MARCOS methodology have been developed base on the FMEA technique. Given the fact that decision-making is essential in the real world, providing effective approaches is critical. For this purpose, in this study, in line with the goals of sustainable development and sustainable mobility, the proposed method was applied to rural roads in the Calabria region in southern Italy. The results showed that most of the risk factors in which drivers had a direct role had the highest level of risk. This study has identified that "lack of driving skills" has the highest priority with 0.291 score among risk factors. Also, "presence of alcohol, medicinal or recreational drugs" and "lack of attention to the longitudinal safety distance" are in the second and third priority with the scores of 0.288 and 0.282, respectively. The proposed method was verified in two ways. As a consequence of these verifications, it was evident that the rating results were reliable, with adequate levels of accuracy and robustness. Based on this, a set of necessary measures to increase the level of awareness of drivers was proposed. The suggested method might be a specific approach but an effective tool for evaluating risk factors impacting rural road safety and, therefore, beneficial in decision-making under uncertainty.

The main weakness of this study was not considering the relations between criteria. The criteria probably have linear or non-linear relations. We may suggest a novel approach for this. In addition, the number of experts was limited. So, increasing the number of experts will change the results to be more reliable. Also, the expert had limited 9 scale linguistic variables to present their opinions. Therefore, increasing the scale of linguistic variables may give them more freedom and increase certainty and confidence. For future research, this study provides directions: the proposed approach can be directly applied to other types of roads or any other issues that related to risk assessment. Additionally, we recommend new aggregation operators to be extended by applying SFS. Another recommendation is to extend other MCDM methods to their spherical fuzzy environments such as BWM, VIKOR, SECA, and TRUST.

## Data Availability

All data generated or analyzed during this study are included in this published article.

## Footnotes

1 The COVID-19 pandemic and the measures adopted to contain it has led to a significant decrease in the traffic flow volumes on the roads, with a consequent decrease in exposure to the accidents risk.
